# Homozygous familial hypercholesterolemia, experience with Evinacumab treatment in two Mexican pediatric patients: case report

**DOI:** 10.3389/fgene.2026.1841116

**Published:** 2026-07-02

**Authors:** Ramón Madriz Prado, Yazmín Guadalupe Ríos Solís, Diana Estefanía Guerrero Dávila, Lucía Molina Fernández, Omar Spencer Aguilar Reyes, Ilse Ordóñez, Leticia Ramos, Marisol González

**Affiliations:** 1 Servicio de Endocrinología Pediátrica, Unidad de Especialidades Médicas, Secretaría de La Defensa Nacional, Mexico City, Mexico; 2 Facultad de Medicina, Universidad Nacional Autónoma de México, Mexico City, Mexico

**Keywords:** case report, cholesterol, Evinacumab, familial hypercholesterolemia, vascular improvement

## Abstract

Homozygous familial hypercholesterolemia (HoFH) is a rare and life-threatening genetic disorder characterized by extremely elevated low-density lipoprotein cholesterol (LDL-C) levels from birth, leading to accelerated atherosclerotic cardiovascular disease and premature mortality. Conventional lipid-lowering therapies often provide insufficient LDL-C reduction, particularly in patients with minimal or absent LDL receptor (LDLR) function. Evinacumab, an angiopoietin-like protein 3 (ANGPTL3) inhibitor, lowers LDL-C independently of LDLR activity and represents a major therapeutic advance. Here we report two Mexican pediatric patients with HoFH who demonstrated profound LDL-C reductions following initiation of Evinacumab (59% and 68% within the first month of treatment), exceeding reductions observed in pivotal clinical trials. Both patients maintained sustained LDL-C reductions during long-term follow-up (up to 22 months). Importantly, temporary treatment interruption in both cases due to administrative and supply related difficulties limited access to Evinacumab was associated with marked rebound hypercholesterolemia. Reinitiation of therapy led to rapid and substantial lipid reduction, demonstrating a clear dechallenge–rechallenge effect and confirming the relevance of a continuous pharmacologic treatment with ANGPTL3 inhibition. Serial vascular imaging in one patient revealed partial regression of subclavian and carotid artery stenosis, as well as reduced aortic wall thickening following sustained LDL-C reduction; adding evidence to the recently described vascular improvement associated with Evinacumab therapy in pediatric HoFH. Both patients also experienced clinically meaningful improvements in quality of life, and treatment was well tolerated without serious adverse events.

## Introduction

Homozygous familial hypercholesterolemia (HoFH) is a rare genetic disorder characterized by markedly elevated LDL-C levels from birth. Lifelong exposure to extreme LDL-C concentrations results in accelerated atherosclerotic cardiovascular disease and, if untreated, leads to premature mortality. This article presents two confirmed cases of HoFH in Mexican pediatric patients, with particular emphasis on early diagnosis and evolving therapeutic strategies, especially the use of Evinacumab, a novel pharmacological agent that has significantly improved clinical outcomes. Baseline demographic, clinical, genetic, and lipid characteristics of both patients are summarized in Table 1.

**TABLE 1:** Baseline clinical characteristics, lipid profile, and prior therapies of the two pediatric patients with HoFH.

Characteristic	Patient 1	Patient 2
Sex	Male	Male
Age at evinacumab initiation	15 years	13 years
BMI	21.7 kg/m^2^	15.5 kg/m^2^
Diabetes/comorbidities	None	None
Age at symptom onset	5 years	3 years
Cutaneous manifestations	Cutaneous xanthomas	Cutaneous xanthomas
Family history	Premature MI in grandfather at 48 years; hypercholesterolemia in both parents and sibling	Maternal grandfather with MI at 55 years; paternal grandmother with hypercholesterolemia
Baseline LDL-C before evinacumab	667–821 mg/dL	773–917 mg/dL
LDL-C immediately before evinacumab	667 mg/dL	575 mg/dL
Baseline total cholesterol	Up to 821 mg/dL	Up to 917 mg/dL
Genetic findings	Biallelic semidominant monogenic LDLR variants: c.325 T>C and c.249delTinsGG	Biallelic semidominant monogenic LDLR variants: c.2037T>A and c.1103G>A
Baseline vascular findings	Subclavian and carotid stenosis; aortic wall thickening	No carotid obstruction; normal cardiac structure/function
Prior lipid-lowering therapies	Cholestyramine (discontinued due to intolerance), high-intensity statin, ezetimibe, LDL apheresis, evolocumab	Rosuvastatin 30 mg/day, ezetimibe 10 mg/day
Response to prior therapies	Inadequate LDL-C control; <15% response to evolocumab	Persistent severe hypercholesterolemia despite therapy
Evinacumab dose	15 mg/kg IV every 4 weeks	15 mg/kg IV every 4 weeks
LDL-C reduction after 1 month	59% reduction	68% reduction

### Case 1

**FIGURE 1:**
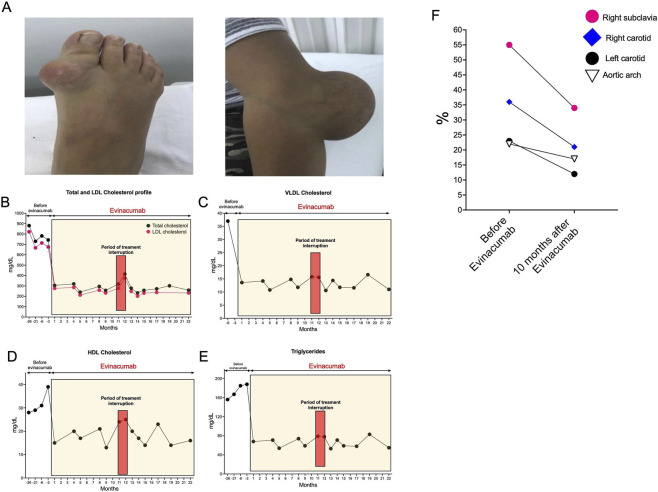
Longitudinal lipid profile in Patient 1 during Evinacumab therapy. **(A)** Presence of cutaneous xanthomas in Patient 1. Shown are the first toe (*left*) and elbow (right). **(B)** Total cholesterol and LDL-C concentrations over time. Measurements are shown before initiation of Evinacumab, during continuous treatment, and during the period of treatment interruption (highlighted in red). A marked reduction in total cholesterol and LDL-C is observed following treatment initiation, with rebound elevation during interruption and subsequent reduction after reintroduction. **(C)** VLDL cholesterol levels during the same follow-up period, demonstrating stabilization during therapy and transient elevation during treatment interruption. **(D)** HDL cholesterol levels across follow-up, showing relative stability throughout treatment and interruption phases. **(E)** Triglyceride levels over time, with reduction after treatment initiation and transient increase during the interruption period. Data are expressed in mg/dL. The shaded area represents the Evinacumab treatment phase, and the red bar indicates the period of temporary treatment interruption. **(F)** Percentage of luminal stenosis in major supra-aortic vessels before initiation of Evinacumab therapy and after 10 months of treatment. A reduction in stenosis severity was observed in the right subclavian artery, right carotid artery, left carotid artery, and aortic arch following treatment.

A 15-year-old Mexican male patient with body mass index (BMI) of 21.7, no diabetes or other preexisting conditions, was initially seen at 5 years of age due to widespread presence of cutaneous xanthomas (Figure 1A) and high cholesterol with blood levels of 821 mg/dL, which according to the European Atherosclerosis Society (EAS) meets the diagnostic criteria for HoFH. Other causes of hypercholesterolemia were ruled out, and the family was examined, discovering that both parents and the brother of the index patient had high cholesterol levels in the blood, and a familial history of premature death (grandfather died at age 48 due to acute myocardial infarction). Presence of multiple early xanthomas and persistently elevated LDL-C levels (>700 mg/dL) prompted the start of cholestyramine therapy, which was discontinued due to adverse events, including meteorism and abdominal pain; therapy was continued with high-intensity statin and ezetimibe treatment. Between 2020 and January 2024 LDL-C levels ranged between 667 and 821 mg/dL. Imaging studies during the same period showed progressive atherosclerotic disease. Molecular test confirmed HoFH due to monogenic biallelic semidominant variants inherited in the *LDLR* (Low-Density Lipoprotein Receptor) gene, namely, LDLR c.325 T>C and LDLR c. 249delTinsGG.

In February 2018, with collaboration from the Mexican Familial Hypercholesterolemia Association the patient was accepted in CEDARS- SINAI Medical Center, United States, where he started weekly apheresis for 6 months, along with high-intensity statin and ezetimibe. Once goal therapy LDL-C levels of 86 mg/dL were achieved, the patient moved back to Mexico in July 2018. Failure to continue apheresis led to a regain of increased LDL-C levels, raising up to 440 mg/dL. The patient was then prompted to start PCSK9 inhibitor therapy (Evolocumab), which however was suspended since reduction of cholesterol levels higher than 15% were not achieved.

### Therapeutic intervention with Evinacumab

In April 2024, this patient started Evinacumab therapy at a dose of 15 mg/kg intravenously administered every 4 weeks. A month after initiation of treatment, LDL-C decreased to 276 mg/dL, a decrease of 59%. After 22 months of continuous treatment, LDL-C levels remained within the range of 200–277 mg/dL. In February 2025, treatment was temporarily interrupted for 1 month due to administrative delays related to drug access and treatment authorization, resulting in an increase in LDL-C levels to 373 mg/dL. Following re-initiation of Evinacumab in March 2025, LDL-C levels decreased to 247 mg/dL and subsequently stabilized within the 200–230 mg/dL range (Figure 1B). Other lipid parameters remained stable during follow-up (Figures 1C–E).

### Vascular outcomes

Contrast-enhanced chest computed tomography (CT) showed a partial regression of vascular stenosis. Right subclavian artery stenosis was 55% and carotid stenosis was up to 32% until treatment, respectively. Long-term reductions in LDL-C levels after Evinacumab therapy led to reduction in right subclavian stenosis up to 34%, 12.8% in left carotid stenosis, and reduction in aortic wall thickening (Figure 1F). These results suggest that sustained reduction in LDL-C was associated with biochemical improvement and measurable structural vascular alterations.

### Quality of life and safety

The WHOQOL scale showed an improvement of 26% overall of perceived quality of life following initiation of treatment. Transient post-infusion headache, which resolved spontaneously, was reported, and no serious adverse events were observe during the 22 months of therapy. The treatment was considered safe and well tolerated. WHOQOL-BREF questionnaires and translated patient responses before and after Evinacumab therapy are provided in Supplementary Appendix S1.

### Case 2

**FIGURE 2:**
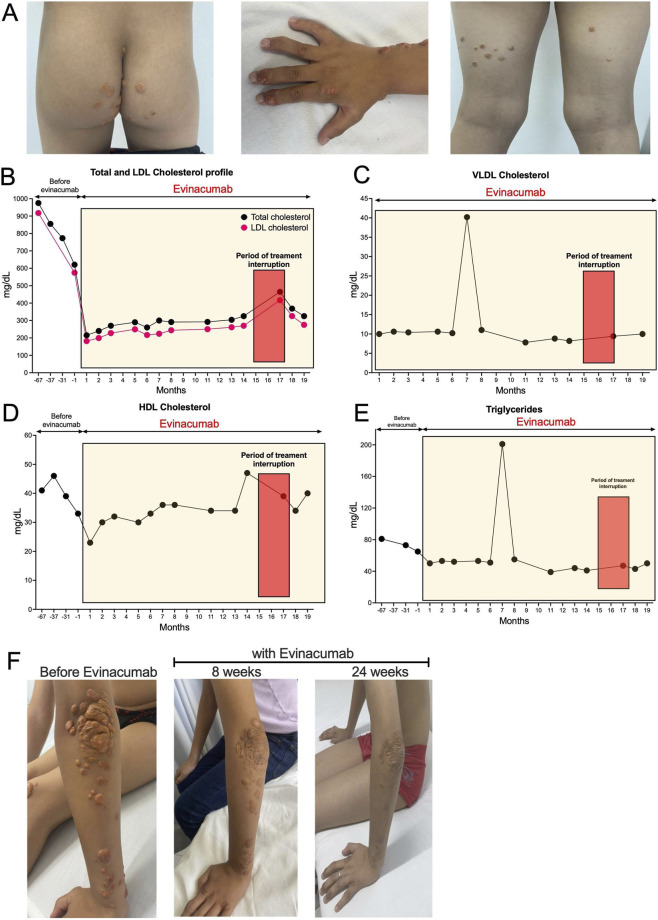
Longitudinal lipid profile in Patient 2 during Evinacumab therapy. **(A)** Presence of cutaneous xanthomas in Patient 2. Shown are the backside of the patient (*left*), right hand (*center*), and inner thighs (*right*). **(B)** Total cholesterol and LDL-C concentrations before and after initiation of Evinacumab. Sustained LDL-C reduction is observed during treatment, with a significant rebound during treatment interruption (red bar) and recovery following reinitiation. **(C)** VLDL cholesterol levels throughout follow-up, showing overall stabilization during treatment and increase during interruption. **(D)** HDL cholesterol concentrations over time, demonstrating modest variation without major fluctuation during treatment. **(E)** Triglyceride levels during follow-up, with improvement under therapy and transient increase during interruption. **(F)** Improvement of cutaneous xanthomas in the elbow and forearm of patient 2. Cutaneous xanthomas before Evinacumab (*Left*). Cutaneous xanthomas 8 weeks after starting treatment with Evinacumab *(Center)*. Cutaneous xanthomas 24 weeks after starting treatment with Evinacumab *(Right)*. Data are expressed in mg/dL. The shaded area indicates the treatment phase with Evinacumab, and the red bar marks the interruption period.

Is a Mexican male patient, who had been under pediatric dermatology follow-up since the age of 5 years and 9 months due to the presence of cutaneous xanthomas that had progressively developed since 3 years of age (Figure 2A). He was subsequently referred for specialized evaluation at 13 years of age. At presentation, his BMI was 15.5 kg/m^2^, with no history of diabetes mellitus or other comorbidities. Laboratory evaluation revealed severe hypercholesterolemia, prompting referral to pediatric endocrinology. A significant family history of premature cardiovascular disease was identified, including a maternal grandfather who experienced a myocardial infarction at 55 years of age requiring coronary revascularization, and a paternal grandmother with hypercholesterolemia. Initial carotid ultrasound in 2018 showed normal vascularity, with no evidence for atherosclerotic plaques. Before any treatment was started, LDL levels were at 917 mg/dL at 5 years old. Despite treatment with rosuvastatin (30 mg a day), ezetimibe (10 mg a day), *P. psyllium* and omega-3, LDL-C levels remained high, ranging between 773 and 917 mg/dL. Molecular test confirmed HoFH due to monogenic biallelic semidominant variants inherited in the *LDLR* (Low-Density Lipoprotein Receptor) gene, namely LDLR c.2037T>A and LDLR c.1103 G>A.

### Therapeutic intervention with Evinacumab

Evinacumab was initiated in July 2024 at 15 mg/kg administered intravenously every 4 weeks. One month after the start of treatment, the LDL-C levels decreased from 575 mg/dL to 182 mg/dL, corresponding to a 68% reduction. LDL-C levels remained between 199 and 270 mg/dL during follow-up. In October 2025, therapy was temporarily discontinued, resulting in an increase in LDL-C levels to 416 mg/dL by November 2025, highlighting a rebound. Following re-initiated therapy in December 2025, LDL-C levels fell again to 325 mg/dL and 275 mg/dL in January 2026 (Figure 2B). VLDL cholesterol (Figure 2C), HDL cholesterol (Figure 2D) and triglycerides (Figure 2E) are also shown. Triglyceride reductions were not observed, likely due to normal baseline levels.

### Vascular outcomes

Cardiac assessment at 13 years of age revealed normal cardiac structure and function without any evidence of carotid obstruction.

### Quality of life and safety

Evaluation via the WHOQOL-BREF scale revealed a 48% increase in perceived quality of life with initiation of treatment and improvement in the cutaneous and tendinous xanthomas (Figure 2F). Evinacumab was well tolerated and it was only reported with rhinopharyngitis. No significant adverse events were observed at follow-up.

### Sustained ANGPTL3 inhibition is required for metabolic control in HoFH

**FIGURE 3:**
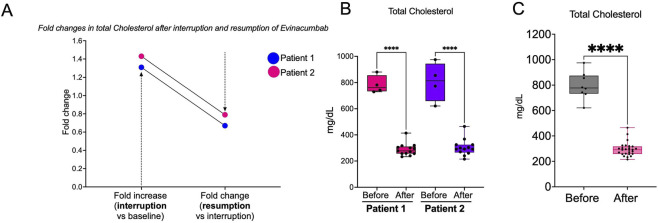
Effect of Evinacumab interruption and reintroduction on total cholesterol. **(A)** Fold changes in total cholesterol following treatment interruption and after resumption of Evinacumab comparing both patients side by side. Interruption resulted in a fold increase in cholesterol levels relative to baseline, whereas reinitiation led to a fold reduction relative to interruption levels. **(B)** Total cholesterol concentrations before and after Evinacumab initiation in each patient. Box plots represent distribution of measurements during each phase. Statistical significance is indicated (****p < 0.0001; ns, not significant). **(C)** Overall comparison of total cholesterol levels in the two reported patients before and after Evinacumab therapy. Treatment was associated with a significant reduction in total cholesterol (****p < 0.0001). Data are expressed in mg/dL. Statistical analysis was performed using paired comparisons.

Of note, both patients experienced temporary interruption of Evinacumab therapy, an unfortunate but frequent challenge in low- and middle-income countries (LMICs), where access to high-cost biologic therapies may be limited. Interruption of Evinacumab therapy resulted in a marked rebound increase in total cholesterol in both patients, with fold increases relative to baseline observed during the dechallenge phase Upon reintroduction of therapy, total cholesterol levels declined substantially, demonstrating a clear rechallenge effect and confirming treatment responsiveness (Figure 3A). Nonetheless, overall response to Evinacumab was sustained after treatment resumption without affecting the ulterior patient’s response. Individual patient analyses showed significant reductions in total cholesterol after initiation of Evinacumab compared with pre-treatment levels (Figure 3B). When both patients were analyzed collectively, Evinacumab therapy was associated with a highly significant overall reduction in total cholesterol (Figure 3C). These findings illustrate a consistent and reproducible dechallenge–rechallenge pattern, supporting the direct and sustained lipid-lowering effect of ANGPTL3 inhibition in HoFH.

## Literature review

The rate of HoFH is estimated to be 1 in 250,000 to 1 in 360,000 people, however, in several nations, especially French Canadians, South African Afrikaners, and Lebanese Christians the prevalence is greater due to founder effects (Cuchel et al., 2023; Defesche et al., 2017). It is estimated that less than 5% of global patients are diagnosed. The most frequent phenotype of this disease is the heterozygous familial hypercholesterolemia, affecting 1 in 250 a 300 people (Cuchel et al., 2023; Corpataux et al., 2025).

### Genetic basis

Familial hypercholesterolemia shows incomplete dominance, with heterozygotes carrying one inherited allele with mild to moderate hypercholesterolemia and the ones associated with homozygotes phenotype (biallelic) exhibiting more severe hypercholesterolemia (Cuchel et al., 2023). This disease is mostly caused by inherited pathogenic variants in four genes: *LDLR* (85%–90% of cases), *APOB* (5%–10%), *PCSK9* (1%–3%), and *LDLRAP1* (<1%). The variants in *LDLR* are categorized as null variants (≤2% residual activity) or defective variants (2%–70% residual activity) based on their functional impact (Cuchel et al., 2023; Iannuzzo et al., 2025). The classification has clinical relevance since null variants are generally associated with higher LDL-C levels.

As highlighted in the updated EAS guides, most patients with HoFH harbor two pathogenic variants either within the same gene (monogenic) or across two different genes (digenic inheritance), and many cases historically referred to as “homozygous” are in fact compound heterozygous. The consensus statement also emphasizes that terms such as “compound heterozygous” and “double heterozygous” may generate confusion and recommends retaining the operational term “HoFH” or “phenotypic HoFH” while acknowledging the underlying genetic complexity. Therefore, 1) HoFH may result from identical or different bi-allelic pathogenic variants in LDLR or other causative genes. 2) Most genetically confirmed cases are compound heterozygotes rather than true homozygotes. 3) Additional rare forms include digenic inheritance involving genes such as *APOB*, *PCSK9*, and *LDLRAP1* (Cuchel et al., 2023; Iannuzzo et al., 2025).

### Diagnostic criteria

Modern standards from the EAS guidelines emphasize phenotypic features over genotypic findings before any clinical suspicion of HoFH. Current HoFH diagnostic criteria consider LDL–C levels >10 mmol/L (>400 mg/dL) highly indicative of HoFH and the most prominent risk factor that warrants further investigations. Additional criteria include a history of cutaneous or tendon xanthomas prior to age 10 and/or maintained elevated LDL-C levels along with heterozygous familial hypercholesterolemia in both parents (Cuchel et al., 2023; Defesche et al., 2017). Confirmatory diagnosis of HoFH relies on the identification of pathogenic or likely pathogenic variants in a biallelic state. A molecular diagnosis may also be established in cases of digenic inheritance, where pathogenic variants are identified in two different genes involved in LDL metabolism (Cuchel et al., 2023).

Once HoFH is suspected, secondary causes of hypercholesterolemia (e.g., nephrotic syndrome, primary biliary cirrhosis, untreated hypothyroidism) should also be ruled out, as other differential diagnosis are sitosterolemia and lysosomal acid lipase deficiency. While clinical diagnosis is emphasized, genetic testing is important since it provides a definitive HoFH confirmation and the opportunity for access to new therapeutic agents and clinical trials, and allows cascade screening for family members (Cuchel et al., 2023).

### Therapeutic objectives and therapeutic management

Lipid-lowering therapy for patients with HoFH should be initiated promptly, aiming to achieve LDL-C targets as defined by international guidelines to effectively reduce cardiovascular risk. The EAS recommends the following LDL-C targets: in healthy adults (≥18 years), <1.8 mmol/L (<70 mg/dL); in adults with established atherosclerotic cardiovascular disease (ASCVD) or additional high-risk features, <1.4 mmol/L (<55 mg/dL); and in children and adolescents (<18 years), <3.0 mmol/L (<115 mg/dL) (Cuchel et al., 2023; Iannuzzo et al., 2025).

In clinical practice, achieving recommended LDL-C targets often requires more than conventional lipid-lowering therapy alone. A stepwise treatment approach should be initiated at diagnosis, beginning with high-intensity statins in combination with ezetimibe. If LDL-C goals are not achieved, treatment should be escalated within approximately 8 weeks to include a PCSK9 inhibitor (e.g., evolocumab or alirocumab). However, the efficacy of PCSK9 inhibitors depends on residual LDL receptor activity and may be substantially reduced in patients carrying null *LDLR* variants (Cuchel et al., 2023; Raal et al., 2020).

Novel therapies that act independently of LDL receptor function have significantly improved the management of HoFH. Lomitapide, an oral microsomal triglyceride transfer protein (MTP) inhibitor, reduces the hepatic production of apolipoprotein B (ApoB)–containing lipoproteins and can achieve LDL-C reductions of up to 60%. Evinacumab, a fully human monoclonal antibody targeting ANGPTL3 (Iannuzzo et al., 2025), provides an additional LDLR-independent therapeutic option. Clinical studies have reported a mean LDL-C reduction of approximately 47% after 24 weeks of treatment in adults with HoFH (Wiegman et al., 2024). In pediatric patients aged 5–11 years, LDL-C reductions of 48.3% have been described, and in an exceptional case involving a 13-month-old infant, an 83% reduction was observed after 8 weeks of therapy (Fornari et al., 2025).

The pivotal ELIPSE HoFH trial demonstrated an approximately 50% reduction in LDL-C levels, independent of underlying LDLR function. Data from both adult and pediatric populations further support the ability of this therapy to achieve substantial and clinically meaningful reductions in LDL-C (Iannuzzo et al., 2025). Evinacumab has also been shown to reduce other atherogenic lipid parameters, including remnant lipoprotein cholesterol (RLP-C) and triglycerides by approximately 50%–88%, small dense LDL-C (sdLDL-C) by 32.2%–37.3%, and several apolipoproteins, including ApoB, ApoC-III, and ApoE by 33.8%–41.4% (Fornari et al., 2025; Matsuda et al., 2025). Evinacumab is indicated as an adjunct to diet and other lipid-lowering therapies in adults and children ≥5 years of age, administered intravenously at a dose of 15 mg/kg every 4 weeks (Cuchel et al., 2023; Ultragenyx México, 2023). Evinacumab has been associated with marked regression of atherosclerotic plaques in adolescents with HoFH, likely driven by sustained reductions in LDL-C levels and the greater biological responsiveness observed in younger patients, whose plaques typically contain less fibrous tissue and calcification (Fornari et al., 2025; Matsuda et al., 2025; Ultragenyx México, 2023; Reeskamp et al., 2021).

Long-term use of Evinacumab has demonstrated sustained efficacy and a favorable safety profile. In patient cohorts followed for up to 24 months after treatment initiation, mean LDL-C reductions of approximately 48% have been reported (Stefanutti et al., 2022), whereas in an Italian cohort, follow-up for 5 years a decrease of up to 50 mg/dL was reported (Iannuzzo et al., 2025). Adverse events were reported in 71.4% of patients, with at least one event occurring in each affected individual; however, only 14% were considered treatment related. The most frequently reported adverse event was oropharyngeal pain (21.4%), followed by upper abdominal pain, diarrhea, nausea, vomiting, headache, and nasopharyngitis (Stefanutti et al., 2022).

### Other therapeutic options

Lipoprotein apheresis is an extracorporeal therapy that remains an important treatment option for patients with HoFH, particularly in countries where newer pharmacological agents are not readily available. The procedure physically removes LDL particles from the circulation and must be performed on a weekly or biweekly basis to maintain efficacy. Current recommendations suggest initiating apheresis as early as 3 years of age and no later than 8 years of age in patients with HoFH (Cuchel et al., 2023). Liver transplantation represents a last-resource therapeutic option for patients who fail to achieve adequate LDL-C control despite maximal pharmacological therapy or who exhibit progressive disease despite optimal management. Since the liver is the primary site of LDL receptor expression, transplantation can restore near-normal LDL-C levels. However, given the substantial perioperative risks, lifelong immunosuppression, and potential complications, it should be reserved as a last-line intervention (Cuchel et al., 2023; Defesche et al., 2017).

## Discussion

HoFH is a severe inherited lipid disorder characterized by markedly elevated LDL-C levels and early atherosclerotic cardiovascular disease. Despite intensive conventional therapy, many patients fail to achieve recommended LDL-C targets due to reduced or absent LDLR activity, highlighting the need for LDLR-independent therapies (Cuchel et al., 2023).

Evinacumab, a monoclonal antibody targeting ANGPTL3 lowers LDL-C via mechanisms unrelated to LDLR function. The important ELIPSE HoFH trial revealed an approximate 49%–50% reduction in LDL-C levels in patients with HoFH, in a manner independent of their underlying genotype (Iannuzzo et al., 2025; Wiegman et al., 2024; Fornari et al., 2025). In the two children described in this report, LDL-C reductions exceeded those observed in clinical trials, reaching 59% and 68% in patient 1 and patient 2, respectively, within the first months of treatment.

In addition, both patients demonstrated sustained LDL-C reductions during long-term follow-up (up to 22 months in patient 1 and up 18 months in patient 2 at the moment of this report). Both patients showed rebound hypercholesterolemia during treatment interruption, which resolved after therapy reinitiation. The observed rebound upon treatment interruption underscores the necessity of continuous ANGPTL3 inhibition to maintain metabolic control in patients with HoFH.

The specific finding of regression of vascular stenosis following sustained LDL-C reduction was especially relevant in patient 1. Serial CT imaging showed improvement of both subclavian and carotid artery stenosis, along with reduced aortic wall thickening. Although large trials have been limited to biochemical endpoints, structural vascular regression has rarely been reported. The long-term and persistent decrease of LDL-C suggests that the early atherosclerotic changes may potentially be partially reversible. Consequently, the long-term effect of LDLR–independent therapy in pediatric patients may be long-term as it can reduce lifetime cardiovascular risk (Reeskamp et al., 2021).

Beyond biochemical and structural outcomes, both patients showed clinically meaningful improvement in quality of life. The WHOQOL evaluations revealed enhanced perceived quality-of-life scores, with 26% improvement in patient 1% and 48% in patient 2. These findings suggest benefits beyond lipid lowering alone.

Evinacumab was well tolerated in the two patients, with no serious adverse events noted during follow-up. Such positive safety data support its use in pediatric populations when necessary.

These two pediatric cases provide meaningful real-world insight into the management of HoFH in Mexican pediatric patients. Evinacumab not only achieved profound and sustained reductions in LDL-C levels—exceeding those reported in clinical trials—but was also associated with regression of vascular stenosis, improved quality of life, and a favorable safety profile. Early diagnosis and timely initiation of LDLR-independent therapies may be critical in modifying the natural history of HoFH, particularly in children with severe phenotypes. Although larger longitudinal studies are required to confirm long-term vascular outcomes, these findings contribute important clinical evidence supporting early and sustained ANGPTL3 inhibition in the treatment of HoFH. The follow up of these two patients also made evident that uninterrupted treatment is critical for patients with HoFH.

## Patient perspective

### Case 1

I am a 16-year-old boy living with Homozygous Familial Hypercholesterolemia. Since I was little, my life was different because I always felt tired, low on energy, and had constant discomfort. I also had small yellow bumps on my skin called xanthomas, especially on my hands, Achilles tendons, and buttocks. Although many people did not understand what they were, to me they were something that made me feel insecure and different.

Some time ago, I started treatment with Evinacumab, and little by little things began to change. One of the most important changes was that the yellow xanthomas started to disappear. Seeing those physical changes gave me a lot of confidence and hope. I have also noticed that I have more energy to do normal activities, spend time with my friends, and concentrate better in school.

Before, I felt exhausted almost all the time. Now I no longer feel that constant fatigue that used to follow me every day. Although the treatment has been positive, I still sometimes experience severe headaches after the infusions or on certain days, but even so, I feel that the benefits have outweighed the difficulties.

The most important thing for me is feeling that I have a chance to live better and take care of my health from a young age. This treatment has not only changed my body, it has also changed how I feel about myself and how I see my future.

### Case 2

I am 13 years old and I am a heterozygous Familial Hypercholesterolemia patient. I am currently receiving an intravenous treatment called “evinacumab,” which has been helping my health a lot because I no longer experience episodes of pain in my head, stomach, and heart. I have also noticed that I no longer vomit or feel dizzy.

Likewise, I have noticed that the “xanthomas” I had on my knees, feet, heels, elbows, buttocks, and hands have visibly decreased. All of this is helping me have a more normal life where I can now wear shoes or sneakers without any discomfort or pain.

In my school activities, I can now write long texts without pain in my fingers caused by holding a pencil. I can also play without fear of hitting or scraping my “xanthomas”; that used to cause me a lot of pain and external bleeding on my skin.

Another physical change I have noticed is that I am growing with each visit for my treatment application, where they also measure and weigh me. I am growing 1–2 cm per month, and that motivates me to continue with my treatment because I want to be tall and strong, but above all, healthy.

## Conclusion

HoFH is a rare inherited lipid disorder associated with exceptionally high morbidity and premature mortality. Early recognition in childhood is critical, as timely diagnosis enables the initiation of therapeutic strategies aimed at altering the natural course of the disease. In this context, Evinacumab represents a major therapeutic advance, demonstrating robust efficacy irrespective of genotype and enabling substantial and sustained reductions in LDL-C levels. Early implementation of LDLR-independent therapies may not only improve biochemical control but also reduce vascular burden and enhance quality of life in pediatric patients with HoFH.

## Data Availability

The raw data supporting the conclusions of this article will be made available by the authors, without undue reservation.
