# Supplementary material for: Homozygous familial hypercholesterolemia, experience with Evinacumab treatment in two Mexican pediatric patients: case report
Source: Front Genet. 2026 Jul 2;17:1841116. doi: 10.3389/fgene.2026.1841116 (PMC13372476; doi:10.3389/fgene.2026.1841116)

# **Supplementary Appendix S1. WHOQOL-BREF Assessments Before and After Evinacumab Therapy**

WHOQOL-BREF questionnaires completed before and after initiation of Evinacumab therapy. Responses are shown in both the original Spanish version completed by the patient and the corresponding English translation. Higher scores indicate improved perceived quality of life.

# **Part A. English Translation**

## **BEFORE TREATMENT**

| **Question** | **Response** |
| --- | --- |
| 1. How would you rate your quality of life? | Very poor (1) |
| 2. How satisfied are you with your health? | Dissatisfied (2) |
| 3. To what extent do you feel that physical pain prevents you from doing what you need to do? | Quite a lot (4) |
| 4. How much do you need medical treatment to function in your daily life? | Completely (5) |
| 5. How much do you enjoy life? | Moderate amount (3) |
| 6. To what extent do you feel your life to be meaningful? | Quite a lot (4) |
| 7. How well are you able to concentrate? | Moderately (3) |
| 8. How safe do you feel in your daily life? | Moderately (3) |
| 9. How healthy is your physical environment? | Quite a lot (4) |
| 10. Do you have enough energy for everyday life? | Moderately (3) |
| 11. Are you able to accept your bodily appearance? | A little (2) |
| 12. Have you enough money to meet your needs? | Moderately (3) |
| 13. How available to you is the information that you need in your daily life? | Moderately (3) |
| 14. To what extent do you have the opportunity for leisure activities? | Quite a lot (4) |
| 15. How well are you able to get around? | Moderately (3) |
| 16. How satisfied are you with your sleep? | Neither satisfied nor dissatisfied (3) |
| 17. How satisfied are you with your ability to perform your daily living activities? | Dissatisfied (2) |
| 18. How satisfied are you with your capacity for work? | Dissatisfied (2) |
| 19. How satisfied are you with yourself? | Neither satisfied nor dissatisfied (3) |
| 20. How satisfied are you with your personal relationships? | Dissatisfied (2) |
| 21. How satisfied are you with your sex life? | Neither satisfied nor dissatisfied (3) |
| 22. How satisfied are you with the support you get from your friends? | Neither satisfied nor dissatisfied (3) |
| 23. How satisfied are you with the conditions of your living place? | Neither satisfied nor dissatisfied (3) |
| 24. How satisfied are you with your access to health services? | Satisfied (4) |
| 25. How satisfied are you with your transport? | Dissatisfied (2) |
| 26. How often do you have negative feelings such as blue mood, despair, anxiety or depression? | Very often (4) |

##

## **AFTER TREATMENT**

| Question | Response |
| --- | --- |
| 1. How would you rate your quality of life? | Very good (5) |
| 2. How satisfied are you with your health? | Very satisfied (5) |
| 3. To what extent do you feel that physical pain prevents you from doing what you need to do? | A little (2) |
| 4. How much do you need medical treatment to function in your daily life? | Completely (5) |
| 5. How much do you enjoy life? | Completely (5) |
| 6. To what extent do you feel your life to be meaningful? | Completely (5) |
| 7. How well are you able to concentrate? | Completely (5) |
| 8. How safe do you feel in your daily life? | Completely (5) |
| 9. How healthy is your physical environment? | Completely (5) |
| 10. Do you have enough energy for everyday life? | Completely (5) |
| 11. Are you able to accept your bodily appearance? | Quite a lot (4) |
| 12. Have you enough money to meet your needs? | Quite a lot (4) |
| 13. How available to you is the information that you need in your daily life? | Completely (5) |
| 14. To what extent do you have the opportunity for leisure activities? | Quite a lot (4) |
| 15. How well are you able to get around? | Quite a lot (4) |
| 16. How satisfied are you with your sleep? | Very satisfied (5) |
| 17. How satisfied are you with your ability to perform your daily living activities? | Very satisfied (5) |
| 18. How satisfied are you with your capacity for work? | Satisfied (4) |
| 19. How satisfied are you with yourself? | Very satisfied (5) |
| 20. How satisfied are you with your personal relationships? | Very satisfied (5) |
| 21. How satisfied are you with your sex life? | Neither satisfied nor dissatisfied (3) |
| 22. How satisfied are you with the support you get from your friends? | Satisfied (4) |
| 23. How satisfied are you with the conditions of your living place? | Very satisfied (5) |
| 24. How satisfied are you with your access to health services? | Very satisfied (5) |
| 25. How satisfied are you with your transport? | Very satisfied (5) |
| 26. How often do you have negative feelings such as blue mood, despair, anxiety or depression? | Seldom (2) |

# **Part B. Spanish Version (Patient Responses)**

## **BEFORE TREATMENT**

| **Pregunta** | **Respuesta** |
| --- | --- |
| 1. ¿Cómo calificaría su calidad de vida? | Muy mala (1) |
| 2. ¿Qué tan satisfecho está con su salud? | Insatisfecho (2) |
| 3. ¿Hasta qué punto piensa que el dolor físico le impide hacer lo que necesita? | Bastante (4) |
| 4. ¿Qué tanto necesita de cualquier tratamiento médico para llevar a cabo su vida diaria? | Totalmente (5) |
| 5. ¿Qué tanto disfruta de la vida? | Moderado (3) |
| 6. ¿Hasta qué punto siente que su vida tiene significado? | Bastante (4) |
| 7. ¿Qué tan capaz es de concentrarse? | Moderado (3) |
| 8. ¿Qué tan seguro se siente en su vida diaria? | Moderado (3) |
| 9. ¿Qué tan saludable es su entorno físico? | Bastante (4) |
| 10. ¿Qué tanta energía tiene para su vida diaria? | Moderado (3) |
| 11. ¿Qué tanto acepta su apariencia física? | Poco (2) |
| 12. ¿Qué tanto su economía le permite cubrir sus necesidades? | Moderado (3) |
| 13. ¿Qué tan disponible tiene la información que necesita en su vida diaria? | Moderado (3) |
| 14. ¿Hasta qué punto tiene oportunidad para realizar actividades recreativas? | Bastante (4) |
| 15. ¿Qué tan capaz es de desplazarse de un lugar a otro? | Moderado (3) |
| 16. ¿Qué tan satisfecho está con su sueño? | Ni satisfecho ni insatisfecho (3) |
| 17. ¿Qué tan satisfecho está con su habilidad para realizar sus actividades de la vida diaria? | Insatisfecho (2) |
| 18. ¿Qué tan satisfecho está con su capacidad de trabajo? | Insatisfecho (2) |
| 19. ¿Qué tan satisfecho está de sí mismo? | Ni satisfecho ni insatisfecho (3) |
| 20. ¿Qué tan satisfecho está con sus relaciones personales? | Insatisfecho (2) |
| 21. ¿Qué tan satisfecho está con su vida sexual? | Ni satisfecho ni insatisfecho (3) |
| 22. ¿Qué tan satisfecho está con el apoyo que le brindan sus amistades? | Ni satisfecho ni insatisfecho (3) |
| 23. ¿Qué tan satisfecho está de las condiciones del lugar donde vive? | Ni satisfecho ni insatisfecho (3) |
| 24. ¿Qué tan satisfecho está con el acceso que tiene a los servicios de salud? | Satisfecho (4) |
| 25. ¿Qué tan satisfecho está con el medio de transporte que utiliza? | Insatisfecho (2) |
| 26. ¿Con qué frecuencia tiene sentimientos negativos, tales como tristeza, desesperanza, ansiedad o depresión? | Muchas veces (4) |

**AFTER TREATMENT**

| **Pregunta** | **Respuesta** |
| --- | --- |
| 1. ¿Cómo calificaría su calidad de vida? | Muy buena (5) |
| 2. ¿Qué tan satisfecho está con su salud? | Muy satisfecho (5) |
| 3. ¿Hasta qué punto piensa que el dolor físico le impide hacer lo que necesita? | Poco (2) |
| 4. ¿Qué tanto necesita de cualquier tratamiento médico para llevar a cabo su vida diaria? | Totalmente (5) |
| 5. ¿Qué tanto disfruta de la vida? | Totalmente (5) |
| 6. ¿Hasta qué punto siente que su vida tiene significado? | Totalmente (5) |
| 7. ¿Qué tan capaz es de concentrarse? | Totalmente (5) |
| 8. ¿Qué tan seguro se siente en su vida diaria? | Totalmente (5) |
| 9. ¿Qué tan saludable es su entorno físico? | Totalmente (5) |
| 10. ¿Qué tanta energía tiene para su vida diaria? | Totalmente (5) |
| 11. ¿Qué tanto acepta su apariencia física? | Bastante (4) |
| 12. ¿Qué tanto su economía le permite cubrir sus necesidades? | Bastante (4) |
| 13. ¿Qué tan disponible tiene la información que necesita en su vida diaria? | Totalmente (5) |
| 14. ¿Hasta qué punto tiene oportunidad para realizar actividades recreativas? | Bastante (4) |
| 15. ¿Qué tan capaz es de desplazarse de un lugar a otro? | Bastante (4) |
| 16. ¿Qué tan satisfecho está con su sueño? | Muy satisfecho (5) |
| 17. ¿Qué tan satisfecho está con su habilidad para realizar sus actividades de la vida diaria? | Muy satisfecho (5) |
| 18. ¿Qué tan satisfecho está con su capacidad de trabajo? | Satisfecho (4) |
| 19. ¿Qué tan satisfecho está de sí mismo? | Muy satisfecho (5) |
| 20. ¿Qué tan satisfecho está con sus relaciones personales? | Muy satisfecho (5) |
| 21. ¿Qué tan satisfecho está con su vida sexual? | Ni satisfecho ni insatisfecho (3) |
| 22. ¿Qué tan satisfecho está con el apoyo que le brindan sus amistades? | Satisfecho (4) |
| 23. ¿Qué tan satisfecho está de las condiciones del lugar donde vive? | Muy satisfecho (5) |
| 24. ¿Qué tan satisfecho está con el acceso que tiene a los servicios de salud? | Muy satisfecho (5) |
| 25. ¿Qué tan satisfecho está con el medio de transporte que utiliza? | Muy satisfecho (5) |
| 26. ¿Con qué frecuencia tiene sentimientos negativos, tales como tristeza, desesperanza, ansiedad o depresión? | Pocas veces (2) |

**PHOTOGRAPHS OF ORIGINAL QUESTIONNAIRES**


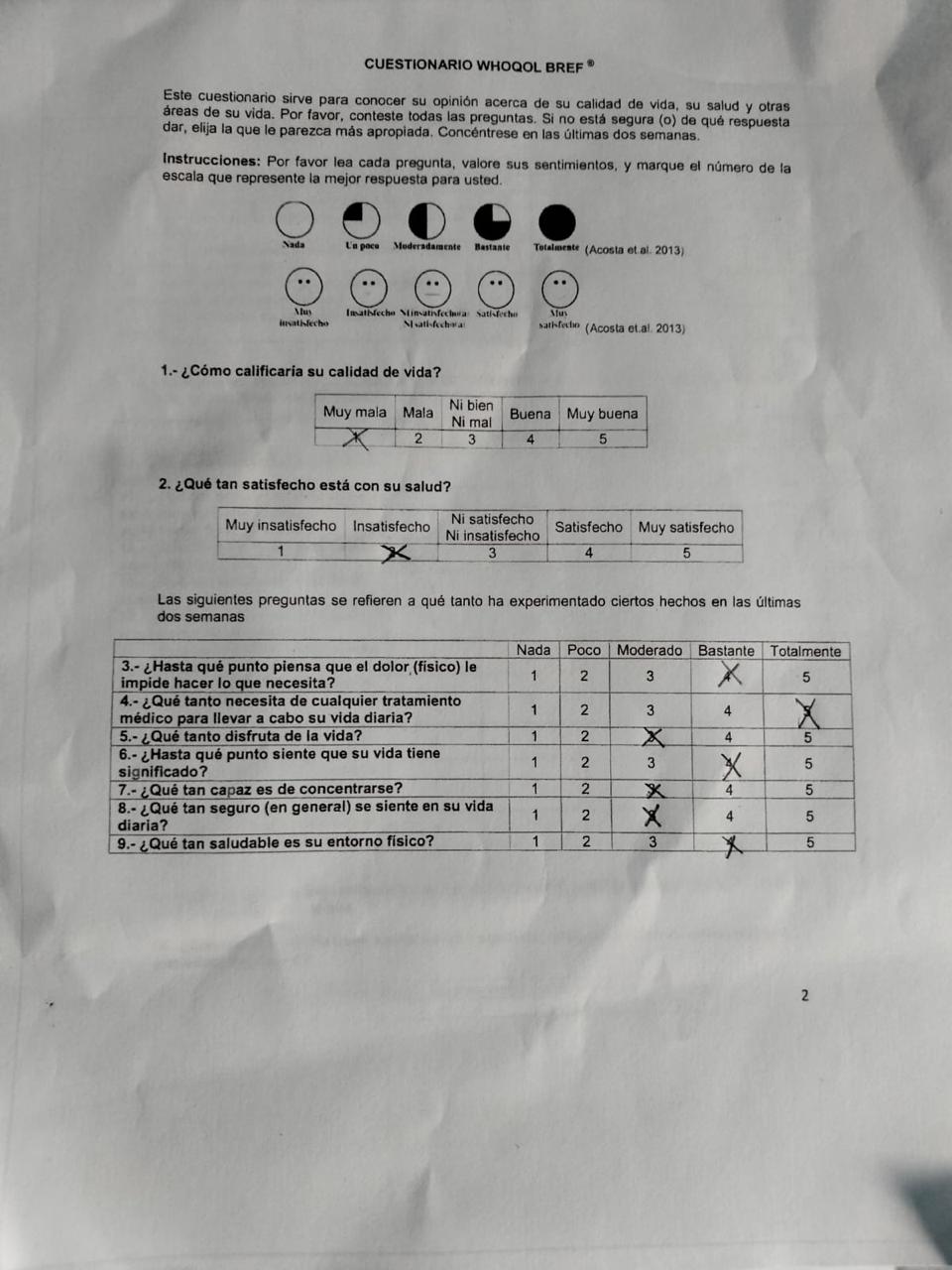


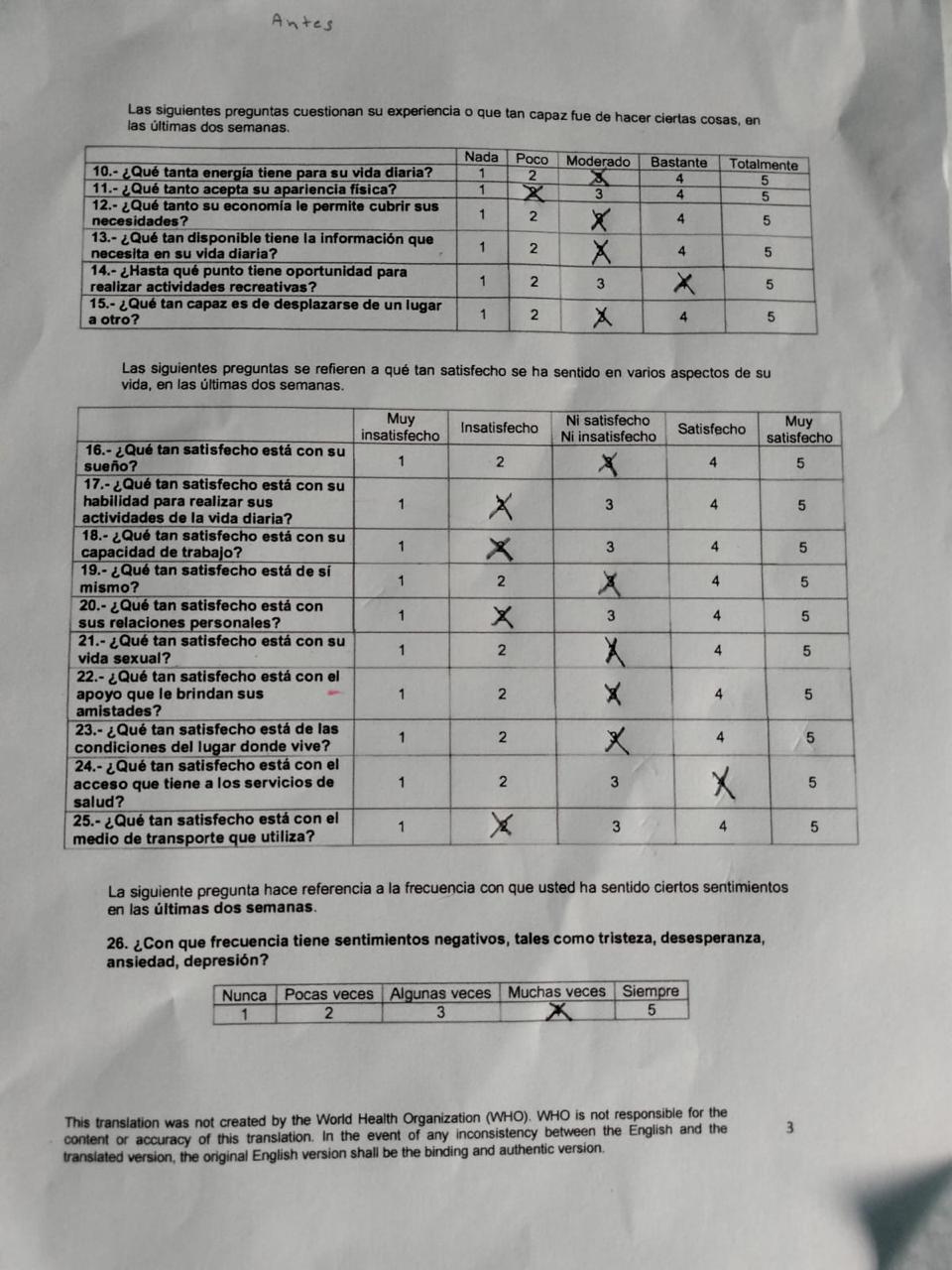


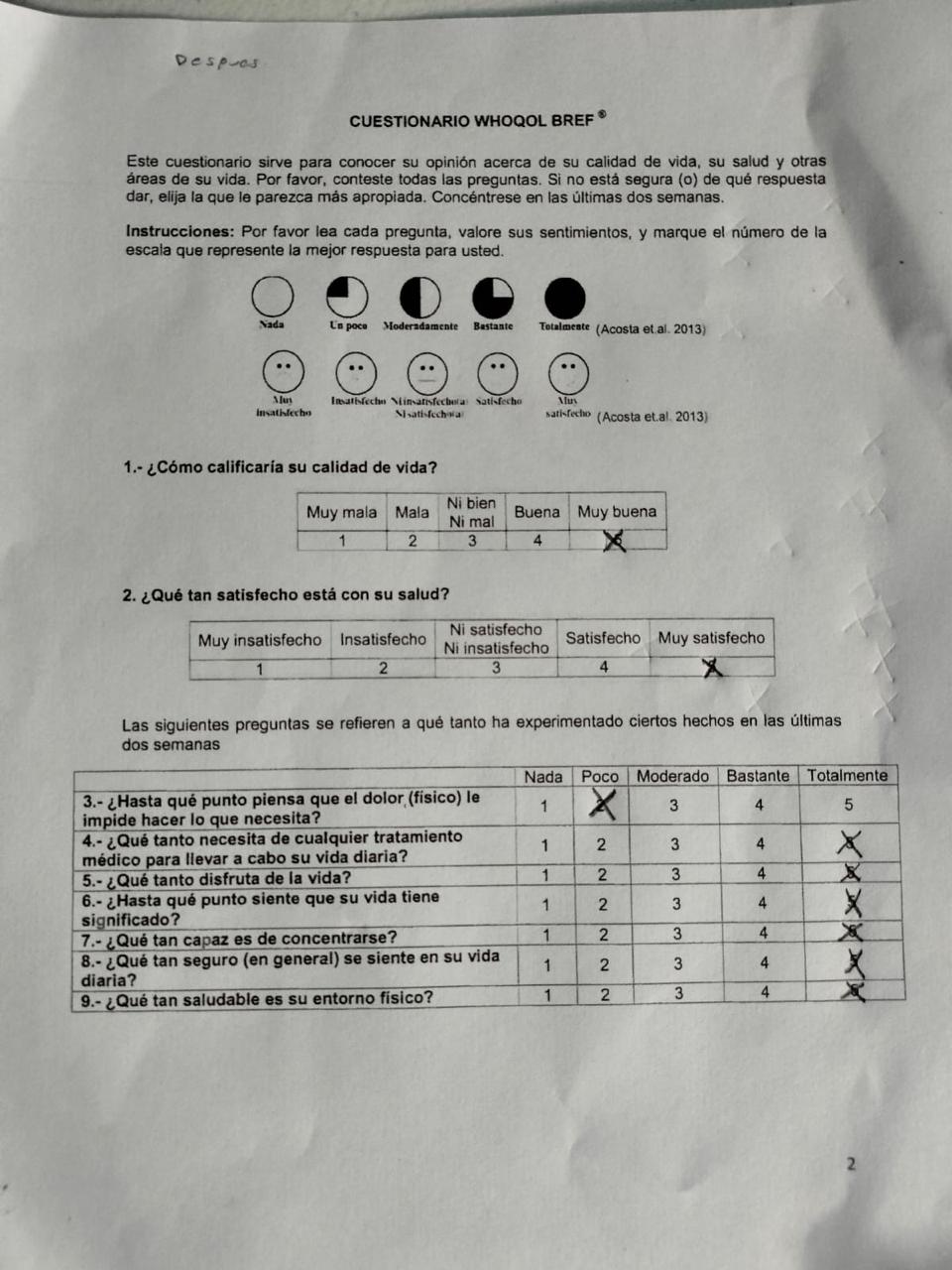


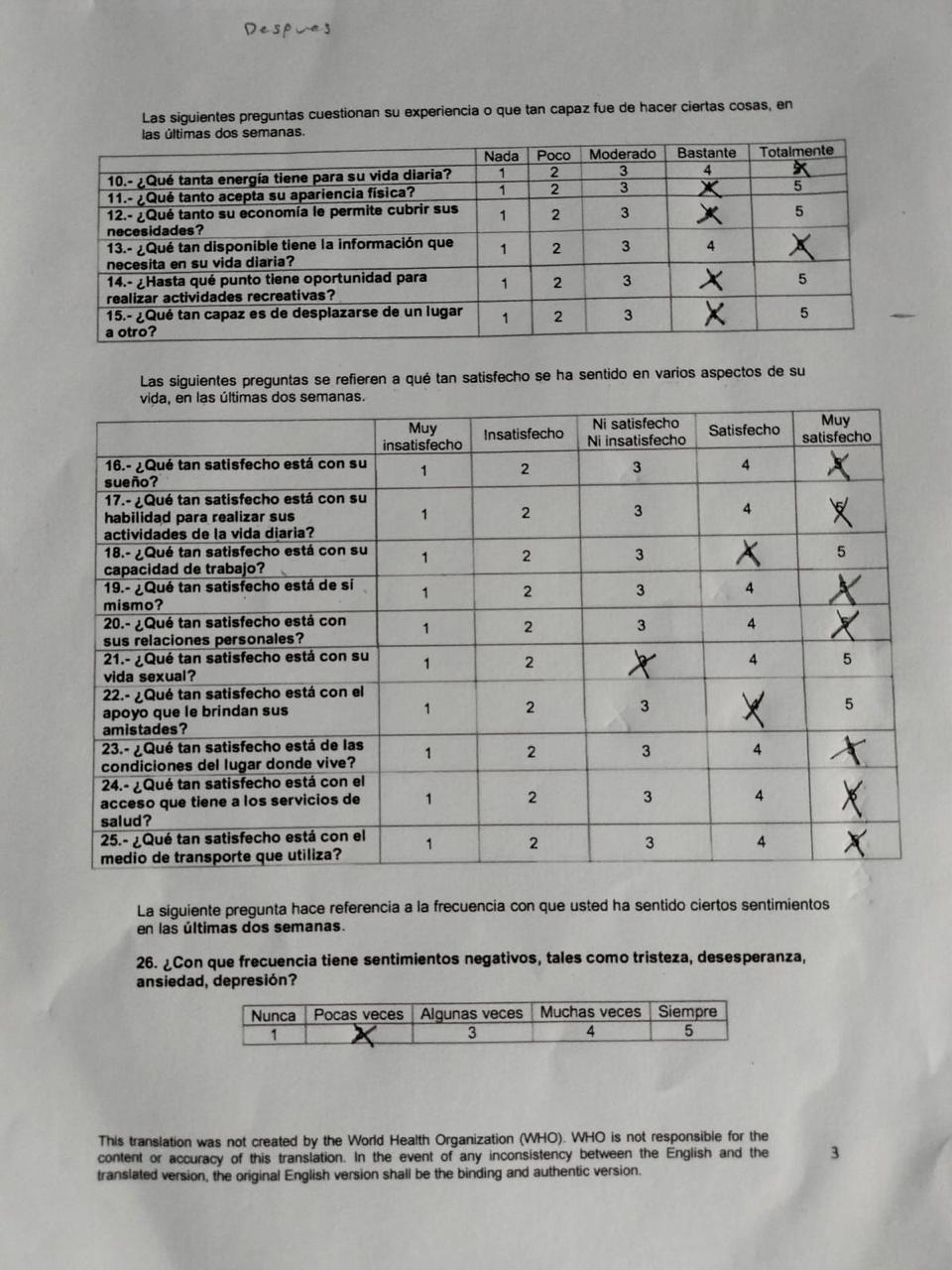

Supplement: Supplementary file 1 [file DataSheet1.docx]
